# Identifying the Challenges of Prehospital Emergency Centers in Providing Care in Wartime and Proposing Strategic Solutions: A Qualitative Study

**DOI:** 10.1002/hsr2.72315

**Published:** 2026-05-10

**Authors:** Jafar Miadfar, Masoumeh Abbasabadi Arab, Hassan Nourisari, Mohammad Sarwar, Reza Dehghanpour, Mostafa Bijani

**Affiliations:** ^1^ National Emergency Medical Organization, Ministry of Health & Medical Education, Prehospital Emergency Research Center Tehran Iran; ^2^ Department of Medical Surgical Nursing, School of Nursing Fasa University of Medical Sciences Fasa Iran

**Keywords:** challenges, emergency medical services, military medicine, qualitative study, wartime

## Abstract

**Background and Aims:**

Emergency Medical Services (EMS) centers, as the primary providers of urgent medical care, play a vital role in preserving lives during crises. Among the most complex and high‐risk scenarios affecting prehospital EMS performance is wartime conditions. Accordingly, the present study sought to identify the principal challenges encountered by prehospital emergency centers in providing care during wartime conditions and to propose strategic, context‐specific solutions aimed at enhancing their effectiveness in Iran.

**Methods:**

This qualitative study examines the difficulties prehospital Emergency Medical Services (EMS) centers in providing care in in Iran faced throughout the war and suggests tactical remedies. 26 participants, including top management and frontline EMS staff, participated in semi‐structured interviews using purposive sampling. Graneheim and Lundman's content analysis methodology was used to examine the data.

**Results:**

Four major themes emerged from the results: (1) Organizational and governance issues (inadequate infrastructure and resources and a lack of thorough wartime procedures), (2) Communication and dispatch issues (lack of wartime telephone triage protocols and GPS disruptions), (3) Professional issues (insufficient competencies in incident scene management and psychological preparedness), and (4) Cultural issues (low public awareness and crowding at incident scenes).

**Conclusion:**

The study comes to the conclusion that the safety, promptness, and efficacy of prehospital emergency care in wartime contexts may be greatly improved by addressing these issues through focused policy formulation, specialized training, enhanced communication systems, and public education. The results are a significant addition to emergency and disaster medicine in Iran and offer useful, context‐specific insights for EMS leaders and policymakers.

## 1 Introduction

Prehospital emergency centers constitute the first point of contact in the provision of urgent medical care and are indispensable in safeguarding lives during emergency situations [1]. Pre‐hospital emergency care is defined as all the emergency medical services that are provided to the patients outside hospitals before the patients are transferred to the nearest medical center [2]. The fundamental mission of EMS centers is to deliver optimal medical care within the shortest possible timeframe. This imperative becomes even more critical in life‐threatening scenarios [3]. The prompt, efficient, and effective delivery of care by prehospital EMS has been shown to markedly reduce injury‐related complications, minimize long‐term disabilities, and ultimately lower mortality rates [4, 5]. Given the inherently unpredictable and high‐pressure nature of their work, EMS personnel are frequently required to manage critically ill or severely injured patients under extreme and often hazardous conditions [6]. In such circumstances, several interrelated factors are essential for ensuring the delivery of safe, high‐quality care within time‐sensitive constraints. These include professional competence, interagency coordination, and cohesive teamwork among emergency response agencies [7].

One of the most demanding and multifaceted contexts for the provision of prehospital EMS is wartime emergency response. In this regard, a study conducted by Quinn et al (2024), in Ukraine revealed that EMS personnel faced multiple challenges, including deficiencies in casualty triage, shortages of essential medical supplies, and significant threats to their psychological well‐being during wartime deployments [8]. Similarly, Sorani et al. (2018) study in Iran identified a range of obstacles confronting EMS providers during disaster situations. These included issues related to infrastructure, information management systems, human resource capacity, administrative coordination, and the overall provision of medical services [9].

## 2 Background in Iran

Iran's first aerial emergency medical service center was established in 2001 in the capital city of Tehran. By August 2022, approximately 3,400 EMS centers were operational nationwide, reflecting a significant expansion of prehospital care infrastructure. These departments consist of urban centers and road centers. Pre‐hospital emergency care personnel have an associate's or bachelor's degree in emergency care. Also, nurses who have a bachelor's or master's degree may be employed in pre‐hospital emergency care. Two staff members must be present in every shift. Due to a lack of staff, the shifts are on a 24‐h basis, and each staff member should work three 24‐h shifts a week. Since the beginning of the US ‐ Israeli missile strikes and bombing airstrikes on Iran, until March 23, 2026, 47 emergency medical bases and 39 ambulances have been destroyed. Also, 51 EMS personnel have been injured.

A review of the extant literature suggests that relatively few studies have qualitatively examined the challenges associated with prehospital emergency care during wartime, particularly within real‐world operational contexts. Furthermore, even fewer investigations have offered practical, context‐specific strategies to mitigate these challenges. This paucity of research underscores the need for a qualitative inquiry, which is particularly well‐suited to capturing the nuanced experiences of EMS personnel and to exploring the multidimensional aspects of emergency care in wartime settings.

Qualitative research is uniquely positioned to elucidate complex phenomena within specific cultural, organizational, and situational contexts. It seeks to interpret events through the lived experiences of those who are directly engaged with the phenomenon under investigation [10]. Fundamentally, qualitative inquiry strives to understand human experience, grounded in the premise that individuals construct reality through personal perception and interpretation. Only by engaging with participants' lived experiences can researchers uncover the underlying meanings and contextual realities that shape those experiences [11]. Moreover, a qualitative approach is instrumental in revealing the intricate operational and structural challenges faced by prehospital EMS centers during wartime and in generating comprehensive, empirically grounded insights into their underlying causes. Without a clear and systematic exploration of the perceptions and experiences of EMS personnel during wartime emergencies, it is not possible to accurately identify core operational challenges or to formulate effective, contextually appropriate strategies for improvement. For these reasons, a qualitative research design was deemed most suitable for achieving the aims of this study. Accordingly, the research team undertook a study entitled “Identifying the Challenges of Prehospital Emergency Centers in Providing Care in Wartime and Proposing Strategic Solutions in Iran.”

## 3 Methods

The present study is a qualitative work of research with a content analysis approach, which is particularly effective for gaining an in‐depth understanding of participants' subjective interpretations of events, individuals, contexts, and lived experiences [12]. In contexts where empirical knowledge is limited regarding how individuals perceive a given phenomenon, a descriptive qualitative framework enables researchers to access nuanced human experiences situated within specific sociocultural settings [13]. The reporting of the study was based on the consolidated criteria for reporting qualitative research (COREQ) checklist [14]. Supporting files: COREQ checklist.

### 3.1 Participants and Data Collection

Data collection was conducted between June and August 2025 through one‐on‐one, semi‐structured interviews. Eligibility criteria required participants to have experience in delivering emergency medical services under wartime conditions caused by US‐Israeli missile strikes and bombing airstrikes on Iran and to express a clear willingness to participate in the study. Individuals were excluded if they exhibited emotional or physical difficulties that rendered them unable to undergo the interview process or if they declined continued participation.

Initially, 42 individuals—including both frontline prehospital emergency personnel and senior EMS administrators—were invited to participate. However, due to scheduling conflicts and the demands of their professional responsibilities, 16 individuals declined. As a result, the final sample consisted of 26 participants: 14 prehospital emergency personnel and 12 senior EMS managers. All participants were selected using purposive sampling with the snowball technique to ensure the inclusion of information‐rich cases. The recruitment process commenced in collaboration with the head of the prehospital emergency department, who identified a candidate likely to provide comprehensive and relevant insights. This initial participant was then asked to recommend a colleague with substantial expertise and direct experience relevant to the study's focus. Recruitment continued until data saturation was achieved, defined as the point at which no new themes or subthemes emerged during successive interviews [15]. Saturation was reached after the 24th interview; however, two additional interviews were conducted to ensure the completeness and confirm the stability of the data.

A total of 26 semi‐structured interviews were conducted. All interviews were held via video calls on WhatsApp, following prior coordination with each participant to establish mutually convenient times. The interviews were conducted in Persian by the corresponding author (MB), an associate professor of nursing with extensive expertise in qualitative research. To ensure linguistic and conceptual accuracy during the reporting phase, the interview transcripts were translated into English by a bilingual translator fluent in both English and Persian, with a deep understanding of the Iranian cultural context. During the expert review phase, a committee comprising the translator and the study's authors systematically compared the English translations with the original Persian transcripts to preserve the intended meanings and cultural nuances. All interviews were audio‐recorded with participants' informed consent. The interview guide was developed according to the views of the research team. The content validity of the questionnaire was examined and verified by 10 professors of nursing and emergency medicine specialist with extensive expertise in qualitative research. Each session lasted between 40 and 55 min. Initial interview questions were broad and exploratory (e.g., “How long have you been working in this field?” and “Could you describe a typical day working during wartime conditions?”). These were followed by more focused questions aligned with the study's central objectives.

To guide the interviews and ensure comprehensive data collection, participants were asked the following core questions:

- From your perspective, what are the principal challenges encountered by prehospital emergency services and personnel in providing care under wartime conditions caused by missile strikes and bombing airstrikes?
- What practical strategies or interventions do you propose to address these challenges?
- What specialized skills or professional competencies are essential for prehospital EMS personnel operating in wartime environments?
- How would you assess the degree of inter‐agency coordination and cooperation between EMS personnel and other emergency response organizations during armed conflicts?

To elicit more detailed responses and deepen understanding, the interviewer employed follow‐up prompts, such as: “Could you elaborate on that?”, “What exactly do you mean by this?”, and “Can you illustrate that point with an example or share a related experience?” These probing questions enabled the researchers to capture rich, contextually grounded data, aligned with the qualitative nature of the inquiry.

### 3.2 Data Analysis Procedure

The qualitative data obtained through interviews were analyzed using conventional content analysis, as outlined by Graneheim and Lundman [16]. To develop a comprehensive understanding of participants' perspectives, each transcript was read multiple times to allow for immersion in the data. The analysis commenced with the identification of meaningful segments—ranging from individual words and phrases to larger textual units—that captured salient issues related to the constraints and operational barriers encountered in aerial emergency medical services. These “meaning units” were subsequently extracted and assigned initial descriptive codes. Following this preliminary coding phase, the research team conducted a comparative analysis to identify patterns, reconcile overlapping codes, and refine discrepancies. Through an iterative process of constant comparison, the codes were grouped into conceptually coherent categories. To ensure analytic accuracy and conceptual integrity, these emergent categories were repeatedly examined in relation to the original transcripts. Once internal consistency and data saturation had been confirmed, broader themes were synthesized through thematic abstraction.

Immediately after each interview, the corresponding author (MB) transcribed the audio recordings and annotated key passages that conveyed notable insights. Each unit of meaning was systematically coded. The primary author (MA) then reviewed the coding for accuracy and confirmed alignment with the underlying data. Codes that demonstrated semantic or conceptual proximity were merged into higher‐order analytical categories. To ensure methodological rigor, the research team met on multiple occasions to critically evaluate the coherence of these categories and to derive the thematic framework. All data management and analysis procedures were conducted using MAXQDA software (version 2007).

### 3.3 Ensuring Rigor and Trustworthiness

To ensure methodological rigor and enhance the credibility of the findings, the study adhered to the evaluative criteria established by Lincoln and Guba [17]. Credibility was reinforced through member checking and peer debriefing. For member validation, both the interview transcripts and corresponding codes were shared with ten EMS professionals, comprising five frontline prehospital care providers and five senior administrators, who were invited to review and verify the accuracy of the coding and interpretations. Simultaneously, a peer review process was implemented involving eight subject matter experts, including four nursing faculty members with expertise in qualitative methodologies and four emergency medicine physicians with relevant research experience. These experts critically evaluated the coding process, thematic categorization, and interpretive strategies, offering validation and constructive feedback. Dependability was supported by a transparent, step‐by‐step account of the analytical procedures, supplemented with illustrative excerpts from the raw audio and textual data. In addition, two members of the research team independently reviewed the analytical outputs and subsequently convened to resolve interpretive discrepancies, thereby ensuring consistency and analytical coherence. Confirmability was achieved through participant validation of the derived themes and subthemes. An audit trail was also maintained, documenting participant demographics, interview conditions, and procedural details, which enhanced the study's transferability. The research team minimized interpretive bias by limiting their engagement with pre‐existing literature prior to the data analysis phase, thereby reducing the risk of confirmatory bias during interpretation. Throughout the data collection and analysis process, bracketing was employed as a methodological safeguard. This entailed the deliberate suspension of researchers' preconceptions, professional expertise, and experiential assumptions to allow for an unmediated representation of participants' lived experiences. The researchers approached the data without imposing external judgments, ensuring that findings authentically reflected participants' narratives. Transferability was further strengthened through rich, contextual descriptions of the research setting, sampling rationale, participant characteristics, and analytic procedures. Verbatim quotations were used to illustrate key findings, thereby enhancing interpretive transparency and enabling readers to assess the relevance and applicability of the results to other contexts.

### 3.4 Ethical Considerations

All participants provided written informed consent prior to their inclusion in the study. The research was conducted in accordance with the principles outlined in the revised Declaration of Helsinki, which delineates ethical guidelines for medical research involving human subjects. Participants were assured of the anonymity and confidentiality of their information. Additionally, the study received approval from the Research Ethics Committees of National Emergency Medical Services, Tehran, Iran (Ethical code: IR. NEMS. REC.1404.004).

## 4 Results

The average age of the participants was 41.04 ± 8.71 years, and their mean work experience was 12.12 ±  5.57 years. Additional demographic characteristics are presented in Table 1. The content analysis yielded 253 codes, which were classified into four themes and eight sub‐themes. Four major themes emerged from the results: (1) Organizational and governance issues (such as inadequate infrastructure and resources and a lack of thorough wartime procedures), (2) Communication and dispatch issues (lack of wartime telephone triage protocols and GPS disruptions), (3) Professional issues (insufficient competencies in incident scene management and psychological preparedness), and (4) Cultural issues (low public awareness and crowding at incident scenes). A comprehensive overview of these themes and sub‐themes can be found in Table 2.

**Table 1:** Demographic information of the participants in the study.

Participants	Gender	Age (year)	Work experience (year)	Education level
P1	Male	58	23	Emergency Medicine Specialist
P2	Male	37	11	Bachelor's degree in Nursing
P3	Male	36	9	Bachelor's degree in Emergency Medicine
P4	Male	53	17	PhD in Health in Disasters and Emergencies
P5	Male	39	8	Master's degree in Nursing
P6	Male	32	7	Bachelor's degree in Emergency Medicine
P7	Male	43	19	Bachelor's degree in Emergency Medicine
P8	Male	48	15	PhD in Health in Disasters and Emergencies
P9	Male	58	21	Emergency Medicine Specialist
P10	Male	38	10	Bachelor of Nursing
P11	Male	42	12	PhD in Health in Disasters and Emergencies
P12	Male	33	8	Bachelor's degree in Emergency Medicine
P13	Male	30	5	Bachelor of Nursing
P14	Male	34	5	Emergency Medicine Specialist
P15	Male	38	14	Bachelor's degree in Emergency Medicine
P16	Male	37	6	Master's degree in Nursing
P17	Male	40	16	Bachelor's degree in Emergency Medicine
P18	Male	49	18	Emergency Medicine Specialist
P19	Male	57	17	Emergency Medicine Specialist
P20	Male	44	15	Emergency Medicine Specialist
P21	Male	39	5	Bachelor's degree in Nursing
P22	Male	33	8	Emergency Medicine Specialist
P23	Male	41	19	Bachelor's degree in Emergency Medicine
P24	Male	49	15	Emergency Medicine Specialist
P25	Male	29	5	Bachelor's Degree in Emergency Medicine
P26	Male	30	7	Emergency Medicine Specialist

**Table 2:** Themes and subthemes extracted from content analysis.

Themes	Sub‐themes
Organizational and governance issues	Inadequate infrastructure and resources Lack of thorough wartime procedures
Communication and dispatch issues	Lack of wartime telephone triage GPS disruptions
Professional issues	Insufficient competencies in incident scene management Psychological preparedness
Cultural issues	Low public awareness Crowding at incident scenes

### 4.1 Organizational and Governance Issues

According to the participants, governance and organizational deficiencies were among the most critical factors impeding the efficiency of prehospital emergency care during wartime conditions. This theme encompassed two subthemes: inadequate infrastructure and resources, and a lack of thorough wartime procedures.

### 4.2 Lack of Thorough Wartime Procedures

From the 18 participants' point of view, the lack of a thorough wartime procedure outlining the procedures for emergency response, personnel, and the coordinated mobilization of auxiliary aid organizations during wartime has resulted in confusion and suboptimal coordination among emergency teams and rapid response units. This lack of structure has, in turn, undermined the efficiency and effectiveness of service delivery by prehospital EMS personnel. One participant articulated this concern as follows:

It is essential that the national Emergency Medical Services (EMS) organization, in collaboration with other aid agencies and informed by the insights of managers and operational staff from prehospital EMS centers, develop a standardized, comprehensive procedure for emergency response and incident command in military and wartime contexts.

(participant 8)

### 4.3 Another Participant Emphasized

In the absence of a standardized, comprehensive, and context‐specific procedures for mobilizing emergency response forces and executing relief operations, task overlap, discretionary decision‐making, and interpersonal conflicts inevitably arise. Everyone ends up acting independently, attempting to assume control of the incident and merely issuing orders.

(Participant9)

### 4.4 Inadequate Infrastructure and Resources

From the 24 participants' point of view, deficiencies in essential infrastructure, specialized equipment, and logistical resources were also identified as a major subtheme within the broader category of governance and organizational challenges. In this regard, one participant observed:

Providing emergency response in wartime conditions is fundamentally different from routine scenarios, as emergency personnel face unpredictable and complex situations such as large‐scale explosions, building collapses, and exposure to toxic or chemical agents. Therefore, specialized equipment tailored for wartime emergency response must be available. Unfortunately, most ambulances are not equipped with CBRNE Kit (Chemical, Biological, Radiological, Nuclear, and Explosive) response kits, which are absolutely critical in incidents involving hazardous substances.

(Participant 18)

### 4.5 Another Participant Stated

In my opinion, one of the most critical and essential infrastructural elements is the establishment and deployment of crisis management warehouses. These facilities are especially vital in wartime conditions. The presence of such crisis management centers during emergencies and armed conflicts can significantly facilitate optimal access to essential resources and emergency response equipment.

(Participant 11)

### 4.6 A Further Point was Raised By Another Respondent

In addition to upgrading and modernizing the ambulance fleet and on‐board medical equipment, it is crucial to anticipate and organize dedicated ambulance service and repair facilities, as well as specialized fuel stations for ambulances during wartime conditions.

(participant 9)

### 4.7 Communication and Dispatch Issues

*This theme comprised two principal components: lack of wartime telephone triage protocols and GPS disruptions*

### 4.8 Lack of Wartime Telephone Triage Protocols

Participants identified the absence of a structured and context‐specific protocol for telephone triage as a principal challenge encountered by prehospital emergency centers in providing care during wartime conditions. One participant remarked:

There is no comprehensive and dedicated protocol for conducting telephone triage in wartime conditions. Dispatch personnel often make errors in both the dispatch process and the remote triage of casualties because they lack standardized guidance and instead rely on personal judgment. This is not necessarily their fault—most have never experienced such conditions and have not received appropriate training. The Training and Operational Deputy Divisions of the National Emergency Medical Services Organization must collaborate with senior managers and prehospital EMS personnel to develop a unified, context‐specific protocol for telephone triage in wartime settings.

(participant 21)

### 4.9 Another Participant Added

It is possible to enhance the competence of dispatch personnel in telephone triage during crises and wartime scenarios through simulation‐based training. Such experiential training can significantly improve their readiness and performance under pressure.

(participant24)

### 4.10 GPS (Global Positioning System) Disruptions

From the 19 participants' point of view, another major challenge related to the communication system, as reported by participants, was the disruption of GPS functionality, network coverage, and radio and wireless communication during wartime operations. One participant explained:

When the GPS system fails or becomes inoperative, accurately locating the site of an incident becomes extremely difficult—especially in the chaotic environment of war. In such situations, it is essential that EMS centers adopt a multilayered, secure, and resilient communication infrastructure to ensure operational continuity.

(participant 15)

Professional issues (insufficient competencies in incident scene management and psychological preparedness

### 4.11 Professional Issues

Another principal theme emerging from the present study was the category of professional issues, which comprised two key subthemes: insufficient competencies in incident scene management and psychological preparedness.

### 4.12 Insufficient Competencies in Incident Scene Management

From the 26 participants' point of view, possessing advanced professional competencies in incident scene management is essential for the effective execution of emergency medical operations, particularly in complex, high‐risk environments such as those encountered during wartime. One participant remarked:

Managing the scene of an incident during war is extremely difficult and complex. If EMS personnel lack the necessary competencies, they will not be able to manage the scene effectively, which leads to disruptions in zoning and victim triage. In such scenarios, one must have sufficient knowledge, essential skills, and the ability to make swift and accurate decisions in order to perform effectively. Otherwise, confusion, disorientation, and cognitive distraction set in, endangering both the responder's and the victim's lives.

(participant 16)

### 4.13 The Participant Continued

I believe it is imperative for EMS administrators to implement structured training programs on incident scene management to empower operational staff within prehospital emergency centers. I recall that during my undergraduate EMS training, there were no dedicated academic courses or practical workshops on incident management in military or wartime conditions. This clearly highlights the need for revisions in the EMS curriculum at the university level.

(participant 5)

### 4.14 Psychological Preparedness

From the 24 participants' point of view, psychological preparedness—particularly stress management, emotional stability, and emotional intelligence—is an essential attribute for emergency medical personnel across all operational contexts, and especially in crisis and wartime environments. In this regard, one participant observed:

One of the most significant challenges faced by EMS personnel—which I have personally encountered on multiple occasions during emergency responses—is the lack of stress management skills under crisis conditions. On one mission during wartime, I was dispatched with a colleague who was extremely anxious and visibly frightened. His hands were trembling, he had no focus, and he was entirely ineffective in the field. I ended up managing the entire patient care process by myself. Honestly, I can't really blame him. When you haven't had prior exposure to working in such high‐stress environments, these reactions are to be expected. It is absolutely necessary to implement continuous and structured training programs in psychological skills for crisis situations. Furthermore, a fundamental revision of the EMS academic curriculum is warranted to incorporate psychological preparedness as a core educational component.

(participant 7)

### 4.15 Another Participant Echoed This Perspective

Many of my colleagues struggle with psychological skills. They become easily agitated, suffer from intense anxiety, emotional exhaustion, and emotional instability. Some even developed post‐traumatic stress disorder (PTSD) as a result of the overwhelming fear they experienced in wartime scenarios. Emergency medical services is an exceptionally demanding, exhausting, and high‐stress profession. Senior managers within the national EMS organization must develop and implement a comprehensive policy and operational framework to support the psychological preparedness of personnel. Moreover, this support should not be limited to EMS staff alone. Their family members also require psychological support and training in stress management. When my family is gripped by fear and anxiety, that emotional distress inevitably transfers to me as an EMS provider and can negatively impact my decision‐making and mental focus.

(participant 12)

### 4.16 Cultural Issues

This theme encompassed two interrelated issues: low public awareness and crowding at incident scenes.

### 4.17 Low Public Awareness

From the 19 participants' point of view, the critical role of public awareness in enhancing the efficiency of emergency response efforts during wartime. One participant noted:

The general public lacks sufficient awareness regarding basic first aid and emergency response during crises and wartime situations. In fact, their unscientific and improper interventions at the scene of an incident can compromise patient safety and, in some cases, even result in death. Given the typically high number of casualties in wartime incidents, public education is crucial. If people are properly trained, they can serve as helpful lay responders and support emergency personnel in managing the situation more effectively. National media, such as the Islamic Republic of Iran Broadcasting (IRIB), should be mobilized to educate the public on crisis and wartime preparedness. Additionally, EMS centers and other aid organizations such as the Red Crescent can play a significant role in improving public knowledge and promoting a culture of first aid and crisis response within the community.

(participant 23)

### 4.18 Crowding at Incident Scenes

From the 18 participants' point of view, the cultural challenge identified in this study was the tendency of civilians to crowd at incident scenes or outside medical facilities, thereby obstructing emergency operations. As noted by participants:

There have been numerous instances in critical situations involving large numbers of casualties where people have gathered at the scene or outside hospitals. Such behavior obstructs the movement of ambulances and significantly disrupts the delivery of emergency services. It appears that public education is essential in this area, and efforts must be made to change public attitudes and behaviors in such situations.

(participant 22)

## 5 Discussion

The present study sought to identify the principal challenges encountered by prehospital emergency centers during wartime operations and to propose strategic, context‐specific solutions aimed at enhancing their effectiveness in Iran. The findings of the present study reveal that the absence of comprehensive procedures for emergency response, along with deficiencies in the mobilization of auxiliary relief organizations during wartime and inadequacies in critical infrastructure, equipment, and resources, constitute major governance and organizational challenges affecting prehospital emergency care in armed conflict settings. These results are consistent with those of Quinn et al. (2024), whose study in Ukraine identified the lack of structured protocols and clinical governance as significant impediments to effective emergency medical response in wartime contexts [8]. Furthermore, a study conducted by Çamcı et al. (2025), identified the ambulance inaccessibility, delays in reaching emergency departments, and widespread shortages of equipment and personnel as significant impediments to effective Gaza's emergency medical services response in wartime contexts [18]. Mohammadi et al., Bijani et al., and Djalali et al. reported that shortages in equipment and resources represent some of the most pressing challenges confronting EMS personnel during disaster and crisis scenarios [19, 20, 21]. Consequently, it is imperative that senior EMS administrators develop and implement strategic plans to ensure the provision of up‐to‐date medical supplies and the deployment of modern, advanced ambulances, thereby enhancing operational efficiency and responsiveness in complex emergencies. These three studies [19, 20, 21] examine the challenges of EMS personnel in disaster relief, and the war situation has not been addressed. Based on the views and experiences of the participants in the present study, relief in war situations is different from disaster relief. The conditions of war are very stressful, daunting, and complicated. The situation is uncontrollable and unpredictable. Suddenly, there is a terrible explosion, a lot of buildings are destroyed, the scene becomes unsafe, and you are faced with a lot of wounded and killed. The blast wave is so severe and terrible that it disrupts the concentration and mental balance of the rescuer and practically disrupts the relief. The fear that the relief environment will be attacked and exploded at any moment, and the ambulance and EMS personnel will be indescribable.

Another principal theme identified in this study pertains to professional issues, including insufficient competencies in incident scene management and psychological preparedness. These findings are corroborated by Sheikhi et al., who similarly observed that inadequate capacity to manage and triage casualties in complex, high‐pressure environments constitutes a critical challenge for EMS personnel [22]. This highlights the necessity for EMS leadership to introduce targeted interventions designed to strengthen field‐based professional skills, including the implementation of specialized training workshops aimed at improving on‐scene management and decision‐making. Furthermore, studies conducted by Hruska et al. and Thielmann et al. demonstrated that EMS personnel operating in unpredictable and high‐stress environments frequently experience acute psychological strain, which may impair clinical judgment and response efficacy [23, 24]. In the present study, from the participants' point of view, EMS personnel faced psychological burden in a conflict setting, including witnessing acts of warfare, elevated stress and anxiety driven by surges in patient volume and acuity, a high influx of severely injured and acutely traumatized patients, including missile attacks. Cumulatively, all of the above factors can result in significant lasting mental health consequences for EMS personnel. Given the inherently stressful and volatile nature of prehospital emergency work, EMS administrators must adopt a comprehensive, evidence‐based approach to enhancing and providing comprehensive psychological support, especially for EMS personnel with family members in military service, are paramount. Such a strategy should incorporate structured training in stress management, emotional intelligence, and emotional stability to safeguard both individual well‐being and clinical performance during crisis response operations. Investing in such initiatives will not only improve the quality of life for EMS personnel but also strengthen the capacity of the EMS system to deliver essential care during response in wartime contexts [25].

The study also identified significant communication and dispatch issues, including a lack of wartime telephone triage protocols and GPS disruptions. These findings align with those of Mohammadi et al., in Iran, and Holmström in Sweden, who reported that the absence of standardized triage protocols, combined with limited training and insufficient expertise among dispatch personnel, constitutes a major professional barrier within EMS communication systems [26, 27]. Similarly, Basnawi et al. found that GPS disruption poses a critical obstacle to effective communication and coordination in EMS settings, thereby reinforcing the findings of the present study [28]. In addition, the analysis revealed two prominent cultural issues: widespread public unawareness in wartime situations and the tendency of civilians to crowd healthcare facilities and incident scenes. In this regard, studies by Hamidizadeh et al. and Bahadori et al. similarly concluded that the general public often lacks a clear understanding of EMS roles and procedures. Their findings indicated that overcrowding and the provision of unscientific or inappropriate first aid—typically arising from insufficient training—can seriously hinder emergency medical operations and, in some instances, endanger patient outcomes [29, 30]. These results underscore the urgent need for targeted public education initiatives focused on first aid and appropriate civilian conduct during emergencies. By promoting a culture of informed engagement and responsible behavior, such programs can enhance the overall efficacy of emergency response efforts in crisis settings.

### 5.1 Limitation

Although the sample size (26 participants) was appropriate for qualitative inquiry, this restricted sampling frame necessarily limits the geographical and institutional transferability of the findings to other provinces or healthcare systems with different organizational cultures and policies. The data in this study were collected over a period of 3 months, which may have beneficial effects on the generalizability of the study results. Therefore, it is recommended that future studies be conducted with longitudinal research over a longer period of time. In addition, future research should prioritize methodological approaches using both quantitative and qualitative approaches to validate and generalize the study results.

### 5.2 Strengths

This study represents the first qualitative investigation in Iran to examine the challenges faced by prehospital emergency medical services in providing care during wartime incidents. Its focus on context‐specific obstacles, combined with the proposal of tailored, practical solutions, constitutes a novel and valuable contribution to the existing body of knowledge in emergency and disaster medicine.

## 6 Conclusion

The study comes to the conclusion that the safety, promptness, and efficacy of prehospital emergency care in wartime contexts may be greatly improved by addressing these issues through focused policy formulation, specialized training, enhanced communication systems, and public education. The results are a significant addition to emergency and disaster medicine in Iran and offer useful, context‐specific insights for EMS leaders and policymakers.

## Author Contributions

Jafar Miadfar, Mostafa Bijani, and Masoumeh Abbasabadi Arab conceptualized and organized the study. Mostafa Bijani and Hassan Nourisari were responsible for data collection and execution. Mohammad Sarwar and Reza Dehghanpour designed and/or executed the statistical analyses. All authors have read and approved the final version of the manuscript. The corresponding author, Mostafa Bijani, had full access to all of the data in this study and takes complete responsibility for the integrity of the data and the accuracy of the data analysis.

## Funding

The authors have nothing to report.

## Conflicts of Interest

The authors declare no conflicts of interest.

## Transparency Statement

The lead author, Mostafa Bijani, affirms that this manuscript is an honest, accurate, and transparent account of the study being reported; that no important aspects of the study have been omitted; and that any discrepancies from the study as planned (and, if relevant, registered) have been explained.

## Supporting information

Supporting File

## Data Availability

The data that support the findings of this study are available from the corresponding author upon reasonable request.
